# Low Capillary Elastic Flow Model Optimization Using the Lattice Boltzmann Method and Non-Dominated Sorting Genetic Algorithm

**DOI:** 10.3390/mi16030298

**Published:** 2025-02-28

**Authors:** Yaqi Hou, Wei Zhang, Jiahua Hu, Feiyu Gao, Xuexue Zong

**Affiliations:** 1 Shanxi Key Laboratory of Chemical Product Engineering, College of Chemistry and Chemical Engineering, Taiyuan University of Technology, Taiyuan 030024, China; 19934921514@163.com (Y.H.); hujiahua816911@163.com (J.H.); gaofeiyu001001@163.com (F.G.); 2 College of Energy and Power Engineering, Lanzhou University of Technology, Lanzhou 730050, China; 17352184398@163.com

**Keywords:** lattice Boltzmann method, microchannel elastic flow, machine learning, NSGA-II, model optimization

## Abstract

In simulations of elastic flow using the lattice Boltzmann method (LBM), the steady-state behavior of the flow at low capillary numbers is typically poor and prone to the formation of bubbles with inhomogeneous lengths. This phenomenon undermines the precise control of heat transfer, mass transfer, and reactions within microchannels and microreactors. This paper establishes an LBM multiphase flow model enhanced by machine learning. The hyperparameters of the machine learning model are optimized using the particle swarm algorithm. In contrast, the non-dominated sorting genetic algorithm (NSGA-II) is incorporated to optimize bubble lengths and stability. This results in a coupled multiphase flow numerical simulation model that integrates LBM, machine learning, and the particle swarm algorithm. Using this model, we investigate the influence of elastic flow parameters on bubble length and stability in a T-shaped microchannel. The simulation results demonstrate that the proposed LBM multiphase flow model can effectively predict bubble elongation rates under complex conditions. Furthermore, multi-objective optimization determines the optimal gas–liquid two-phase inlet flow rate relationship, significantly mitigating elastic flow instability at low capillary numbers. This approach enhances the controllability of the elastic flow process and improves the efficiency of mass and heat transfer.

## 1. Introduction

Microchannel technology, a foundational component in the field of microfluidics, is extensively applied across various sectors, including chemical engineering [1], environmental monitoring [2], and biomedicine and drug delivery [3]. In microreactor research, elastic flow (also known as Taylor flow) has gained increasing prominence due to its potential to enhance heat and mass transfer efficiency. In such systems, bubble flow should feature a sizable gas–liquid contact surface to facilitate efficient heat and mass transfer between the gas and liquid phases, thereby promoting greater exchange of solutes and heat and improving overall transfer capacity [4]. Additionally, the generation and development of bubbles should be highly dispersed, with each bubble segment evenly spaced to maintain uniformity [5,6]. To fully exploit the advantages of elastic flow, considerable emphasis has been placed on achieving uniform bubble generation and consistent bubble size. However, when bubbles become larger, the system may experience non-uniform bubble formation, which can decrease heat and mass transfer efficiency due to the variation in bubble size and surface area [7,8]. This non-uniformity can also lead to undesirable phenomena, such as the agglomeration and splitting of the liquid within the channel [9], compromising the system’s performance. Therefore, controlling bubble size and uniformity is crucial for optimizing the application of elastic flow in microchannel systems.

T-shaped microchannels are widely used to generate Taylor flow, essential for numerous microfluidic applications. In the context of bubble size control, Garstecki [10] and Korczyk [11] explored the complex relationship between bubble length and various factors, such as flow rate, viscosity, and interfacial tension, under steady-state conditions, and established relevant equations. However, the behavior of bubbles in unsteady flow remains less understood. To generate uniform and controllable bubble sizes in T-shaped microchannels or microreactors, Zaloha [12] utilized the v-PIV technique to establish the circulating velocity field inside the bubbles. Zaloha’s work highlighted that bubble elongation was primarily due to asymmetric circulating motions within the bubbles. However, the complexity of the governing parameters, the effects of surface roughness, and the compressibility of the gas phase prevent further refinement of the model.

Currently, multiphase flow simulation in microreactors is developing towards high accuracy, multi-scale, and engineering practicality while relying on interdisciplinary approaches (e.g., mesoscopic simulation, machine learning) to address the limitations of traditional models. Li [13] achieved momentum transfer between fluid and solid particles and two-way real-time coupling of pressure and velocity fields by combining CFD and DEM methods and introduced ultrasonic excitation, which significantly improved the flow distribution and particle homogeneity in microchannels and suppressed particle aggregation and channel clogging; Zheng [14] established a fully resolved reactive particle model and applied dissipative particle dynamics (DPDs) to capture the bubble behavior and applied an efficient parallel algorithm for multiphase flow to improve the accuracy and computational efficiency, which promoted the refinement of the phase change and flow coupling; Dangla et al. [15] improved the pipeline structure to minimize the effect of flow velocity variations on the bubble development, which improved the overall bubble behavior to some extent. Despite the refinement of research in microchannels in recent years, there are still some problems in microchannel elastic flow, and theoretical complexity is still an obstacle in determining the optimal parameters. Peng et al. [16] conducted many repetitive experiments in T-shaped microchannels, changing the flow parameters and establishing a bubble pressure model for unsteady flow. Although the model described the pressure trends in liquid phase systems such as water and ethanol, it did not result in optimal flow parameters, the choice of flow parameters lacked consistency, and the prediction accuracy for different gas–liquid systems and equipment configurations remained problematic.

The lattice Boltzmann method (LBM) has become an effective tool for studying the microscopic flow of multiphase fluids. Based on the Boltzmann equation, the LBM transforms the flow of a continuous medium into discrete particle motion and is, therefore, well-suited for solving complex partial differential equations [17]. This feature enables the LBM to capture detailed information about microscopic interfaces and fluid dynamics [18]. The LBM has been successfully applied in the fields of fuel cells [19], fine chemistry [20,21], carbon capture [22], and wastewater treatment [23].

When applying the lattice Boltzmann method (LBM) to simulate elastic flow at low capillary numbers, selecting the appropriate flow parameters (such as fluid flow rate, viscosity, and interfacial tension) to generate uniform and stable large bubbles is challenging due to the complexity of the bubble development process. Improper parameter selection can lead to non-uniform and tiny bubbles, negatively affecting the precise control of heat, mass transfer, and reaction efficiency in microreactors, resulting in lower conversion rates. It is important to note that machine learning models can predict multiphase flow processes without requiring complex fluid dynamics analysis. For example, Shafizadeh [24] applied Gaussian process regression (GPR), support vector regression (SVR), and other models in the field of hydrothermal liquefaction of biomass to determine the optimal ratio of reaction materials and conditions. Similarly, Bandi et al. [25] expanded this approach by incorporating linear regression (LR), random forest (RF), and artificial neural network (ANN) models to address challenges in evaluating complex computational indices. By integrating the LBM multiphase flow simulation results with machine learning models, parameters such as fluid physical properties (e.g. viscosity, density, interfacial tension) can be input. At the same time, bubble elongation serves as the output for modeling. This approach enables the identification of optimal combinations of parameters to achieve uniform and stable flow conditions, thus overcoming the difficulties associated with the current LBM elastic flow simulations. Additionally, since large bubbles are often associated with superior heat and mass transfer efficiency but tend to disrupt flow uniformity, non-dominated sorting genetic algorithms (NSGA-II) can be used for multi-objective optimization. This method identifies the Pareto optimal solution set, balancing bubble size maximization with steady-state flow stability.

This paper’s original data are generated by simulating the LBM multiphase flow model under various parameter conditions. These data are then fed into a machine learning model for further analysis. Given the nonlinear and complex relationships between parameters and the limited amount of simulation data, machine learning models with strong fitting capabilities and robust performance are essential. Therefore, this study utilizes backpropagation (BP), support vector regression (SVR), and random forest (RF) models, which are well-suited for handling high-dimensional data and nonlinear relationships. Due to numerous hyperparameters in the coupled machine learning–elastostatic flow model and the fact that these hyperparameters cannot be directly learned from the data, the particle swarm optimization (PSO) algorithm is incorporated to optimize the machine learning model. This establishes an LBM–machine learning–particle swarm optimization coupled multiphase flow numerical simulation model. The bubble size and uniformity are incorporated into the non-dominated sorting genetic algorithm (NSGA-II) for multi-objective optimization to enhance the optimization process further, thereby identifying the Pareto front. The coupled numerical simulation model developed in this study addresses the issue of flow instability at low capillary numbers, improving the controllability of the elastic flow process and enhancing the efficiency of mass and heat transfer.

## 2. LBM Multiphase Flow MCMP Model and Validation

### 2.1. MCMP Model

The gas–liquid two-phase flow lattice Boltzmann method (LBM) model evolved from the Lattice Gas Automata (LGA) model in the 1970s. Over nearly 50 years of continuous research by numerous scholars, the model transitioned from the LGA framework to the foundational LBM structure. This development was further advanced with the Chapman–Enskog (C-E) expansion proposal, enabling macroscopic equations to recover. Subsequently, various two-phase flow models have been proposed, with the most commonly used being the DdQm model introduced by Qian et al. [26]. In this model, the motion of the fluid is described by a set of particle distribution functions, enabling flow calculations in the gas–liquid large-density-ratio regime. Due to its discrete lattice-based structure, boundary conditions can be easily applied directly to the lattice, eliminating the need for complex mesh generation and boundary fitting. As a result, the method is more convenient and flexible in handling complex boundary conditions.

The standard LB equation without the force term can be expressed as Equation (1):

(1) $$f_{\sigma ,\alpha }\left(x+e_{\alpha }\Delta t,t+\Delta t\right)-f_{\sigma ,\alpha }\left(x,t\right)=-\frac{1}{\tau_{\sigma }}\left(f_{\sigma ,\alpha }\left(x,t\right)-f_{\sigma ,\alpha }^{eq}\left(x,t\right)\right)$$

fσ,i(x,t) is the distribution function and f_eq_σ,i(x,t) is the equilibrium distribution function as in Equation (2):

(2) $$f_{\sigma ,\alpha }^{eq}=\omega_{\alpha }\rho_{\sigma }\left[1+\frac{e_{\alpha }\;\cdot \;u_{\sigma }^{eq}}{c_{s}^{2}}+\frac{{\left(e_{\alpha }\;\cdot \;u_{\sigma }^{eq}\right)}^{2}}{2c_{s}^{4}}-\frac{u_{\sigma }^{eq2}}{2c_{s}^{2}}\right]$$

The left side of the equation represents the flow term, and the right side represents the collision term. This paper discusses the commonly used D2Q9 model, where e_α_ is the lattice discrete velocity and cs is the lattice speed of sound. Usually, c_s_ is taken to be 1/√3 times the speed of sound and $u_{\sigma }^{eq}$ is the velocity vector of the component σ fluid, Equation (3):

(3) $$e_{\alpha }=\left\{\begin{array}{cc} 0 & \alpha =0\\ \left(\cos\left[\frac{\pi \left(\alpha -1\right)}{2}\right],\sin\left[\frac{\pi \left(\alpha -1\right)}{2}\right]\right), & \alpha =1,2,3,4\\ \sqrt{2}\left(\cos\left[\frac{\pi \left(\alpha -5\right)}{2}+\frac{\pi }{4}\right],\sin\left[\frac{\pi \left(\alpha -5\right)}{2}+\frac{\pi }{4}\right]\right), & \alpha =5,6,7,8 \end{array}\right.$$

ω is the weight of D2Q9, ω_0_ = 4/9, ω_1-4_ = 1/9, and ω_5-8_ = 1/36. τ_σ_ is the relaxation time corresponding to the component σ, which is calculated from the kinematic viscosity as in Equation (4), where v_σ_ stands for the kinematic viscosity of the component σ:

(4) $$\tau_{\sigma }=\frac{\nu_{\sigma }}{c_{s}^{2}}+0.5$$

In the Shan–Chen model, in order to introduce non-localized forces, the force on the x position component σ subjected to is defined as follows:

(5) $$\begin{array}{c} F_{\sigma }\left(x\right)=-\varphi_{\sigma }\left(x\right)G_{\sigma \bar{\sigma }}\sum_{\alpha =1}^{N}\;\omega \left({\left|e_{\alpha }\right|}^{2}\right)\;\varphi_{\bar{\sigma }}\left(x+e_{\alpha }\Delta t\right)e_{\alpha }\\ -\varphi_{\sigma }\left(x\right)G_{\sigma \sigma }\sum_{\alpha =1}^{N}\;\omega \left({\left|e_{\alpha }\right|}^{2}\right)\;\varphi_{\sigma }\left(x+e_{\alpha }\Delta t\right)e_{\alpha } \end{array}$$

Although the conservation of mass is satisfied, there is a momentum error when there is an action force, so Guo et al. [27] proposed a new action force format to eliminate the error:

(6) $$F_{\alpha }=\left(1-\frac{1}{2\tau }\right)\omega_{\alpha }\left[\frac{e_{\alpha }-u}{c_{s}^{2}}+\frac{\left(e_{\alpha }\;\cdot \;u\right)}{c_{s}^{4}}e_{\alpha }\right]F$$

$G_{\sigma \sigma }$ It denotes the strength of the attraction between the same components and $G_{\sigma \bar{\sigma }}$ the strength of the repulsion between different components, which naturally separates the phase interfaces by introducing G without needing to track the phase interfaces. This force can be viewed as a pairwise pointwise coupling between the effective mass at position x and the effective mass at the neighboring position $x+e_{i}∆t$. It complies with Newton’s third law and is globally mass-conserving. This interaction force acts as an external force during the collision. It cannot maintain local momentum conservation, but since the system momentum is conserved, the total force acting on the particle at x is the sum of the forces acting in all directions. φ_σ_ is the effective mass, and its expression is as follows:

(7) $$\varphi_{\sigma }=\rho_{\sigma 0}\left[1-e^{-\frac{\rho_{\sigma }}{\rho_{\sigma 0}}}\right]$$

The equilibrium distribution velocity of fluid particles is changed by adding the action force through the Shan–Chen scheme, which is also known as a velocity-displacement-force scheme, which is improved by the Guo format, which is consistent with the macroscopic N-S equation and overcomes the discrete effect error. However, it sacrifices part of the model stability, and this paper is mainly to study the multicomponent flow, so it introduces the mixing average velocity as in Equation (8):

(8) $$u_{\sigma }^{eq}=\frac{\sum_{\sigma }\frac{\rho_{\sigma }u_{\sigma }}{\tau_{\sigma }}}{\sum_{\sigma }\frac{\rho_{\sigma }}{\tau_{\sigma }}}+\tau_{\sigma }\Delta t\frac{F_{\sigma }}{\rho_{\sigma }}$$

The fluid density and the fluid mixing velocity can be obtained from the first-order and second-order moments of the density distribution function, respectively, and based on this, the improved Guo scheme calculates the density and mixing velocity:

(9) $$\rho_{\sigma }=\sum_{\alpha =1}^{N}f_{\sigma ,\alpha }$$

(10) $$\rho \;u=\underset{\sigma }{\sum }\;\rho_{\sigma }u_{\sigma }+\frac{\Delta t}{2}\underset{\sigma }{\sum }\;F_{\sigma }$$

### 2.2. Multi-Relaxation MRT-Guo Format

Compared to the single relaxation time (SRT) model, the multiple relaxation time (MRT) model offers improved numerical stability by reducing the risk of oscillations and dispersion. This is achieved by introducing multiple relaxation times and performing independent relaxation processes for different moments, allowing for a more accurate simulation of complex flows. Furthermore, by adjusting these relaxation times, the MRT model can better approximate the physical properties of real fluids, thus enhancing the accuracy and reliability of the simulation results. In this paper, the MRT-Guo formulation is utilized, and its evolution equation is given by Equation (11):

(11) $$f_{\alpha }\left(x+e_{\alpha }\Delta t,t+\Delta t\right)=f_{\alpha }\left(x,t\right)-{\left(M^{-1}SM\right)}_{\alpha \beta }\left(f_{\beta }\left(x,t\right)-f_{\beta }^{eq}\left(x,t\right)\right)+S_{\alpha }^{\prime }\left(x,t\right)$$

where M is a 9 × 9 transformation matrix:

(12) $$M=\left[\begin{array}{ccccccccc} 1 & 1 & 1 & 1 & 1 & 1 & 1 & 1 & 1\\ -4 & -1 & -1 & -1 & -1 & 2 & 2 & 2 & 2\\ 4 & -2 & -2 & -2 & -2 & 1 & 1 & 1 & 1\\ 0 & 1 & 0 & -1 & 0 & 1 & -1 & -1 & 1\\ 0 & -2 & 0 & 2 & 0 & 1 & -1 & -1 & 1\\ 0 & 0 & 1 & 0 & -1 & 1 & 1 & -1 & -1\\ 0 & 0 & -2 & 0 & 2 & 1 & 1 & -1 & -1\\ 0 & 1 & -1 & 1 & -1 & 0 & 0 & 0 & 0\\ 0 & 0 & 0 & 0 & 0 & 1 & -1 & 1 & -1 \end{array}\right]$$

The lattice Boltzmann method (LBM) employed in this paper models the microscopic behavior of a fluid by tracking the distribution function across a discrete lattice of points. In this context, M represents a 9 × 9 transformation matrix and is a diagonal relaxation matrix:

S = diag(τρ^−1^,τe^−1^,τξ^−1^,τj^−1^,τq^−1^,τj^−1^,τq^−1^,τν^−1^,τν^−1^), physical quantities within the matrix for different relaxation times, τρ^−1^ and τj^−1^; to satisfy the conservation of mass and momentum one should take 1, τν-1 and τe-1 for determining kinematic and bulk viscosity; this paper selects τξ^−1^ = 1.1, τq^−1^ = 1.2; Sa′ is the force term in the velocity space. The LBM evolution equations satisfy the mass conservation, but a momentum error exists when an action force exists. Guo et al. [27] proposed a new action force format to eliminate errors.

Both sides of the evolution equation are multiplied by M to obtain the MRT-LB equation as Equation (13):

(13) $$m^{*}=m-S\left(m-m^{eq}\right)+\Delta t\left(I-\frac{S}{2}\right)\;S_{f}$$

m* is the distribution function in the moment space after migration, and m and meq can be obtained by multiplying M by f and feq, respectively. I is the unit matrix, and Sf is the force term at the moment space.

The computational steps are to initialize the relaxation time and set the initial conditions, calculate m, m_eq_, and S_f_ after collision and migration, and finally obtain the post-collision density function f by inverse operation.

### 2.3. Atmospheric Liquid Density Ratio CS-EOS Equation of State

To achieve larger gas–liquid density ratios and overcome the limitations of the original Shan–Chen model, Yuan et al. [28] enhanced the model’s performance by incorporating various non-ideal gas equations of the state into the pseudopotential framework. These include, for example, the van der Waals equation (vdw), Redlich–Kwong (R-K), Carnahan–Starling (C-S), and Peng–Robinson (P-R) equations. In this paper, the Carnahan–Starling equation of state (CS-EOS) is employed, and the non-ideal equation of state derived from the original Shan–Chen formulation is given by Equation (14):

(14) $$p=\rho c_{s}^{2}+\frac{g}{2}{\left[\varphi \left(\rho \right)\right]}^{2}$$

where ρ represents the fluid density and φ(ρ) is a function of the effective density, and different EOS can be coupled by changing the expression of the effective density φ. The CS-EOS is given in Equation (15).

(15) $$p_{EOS}=\rho RT\frac{1+\frac{b\rho }{4}+{\left(\frac{b\rho }{4}\right)}^{2}-{\left(\frac{b\rho }{4}\right)}^{3}}{{\left(1-\frac{b\rho }{4}\right)}^{3}}-a\rho^{2}$$

The Equation (15) parameters are set as a = 1, b = 4, and R = 1. Gas–liquid separation occurs when the temperature is below the critical temperature, enabling phase coexistence at the same temperature. By lowering the temperature, the model’s density ratio can be improved. However, the original Guo formulation is unsuitable for calculations at temperatures lower than 0.8TC due to numerical stability issues. This limitation was addressed by Li et al. [29], who introduced the parameter ε into the model to achieve thermodynamic consistency and allow for the calculation of the atmospheric liquid density ratio under the Guo format. This modification was later extended to the MRT format [30].

### 2.4. Boundary Conditions and Unit Conversions

Boundary conditions are a critical component of the lattice Boltzmann method (LBM), as they significantly influence model quality, simulation accuracy, and the stability and convergence of numerical calculations [31]. This section focuses on the implementation of flat boundary conditions. Additionally, LBM calculations are performed in lattice units, which must be converted to macroscopic units when addressing real-world physical problems. In this study, the half-bounce format is applied to model the virtual wall of the main channel. This approach positions the solid boundary at the midpoint of the lattice rather than on the lattice point itself, with particles reflecting off the wall after a time step of Δt/2, thus reducing the time step by one compared to the standard bounce while maintaining second-order accuracy [32]. For the static droplet test model, periodic boundary conditions are applied to all four boundaries. At the same time, the exit employs a fully developed boundary with zero directional derivatives for the exit variables.

**Figure 1:**
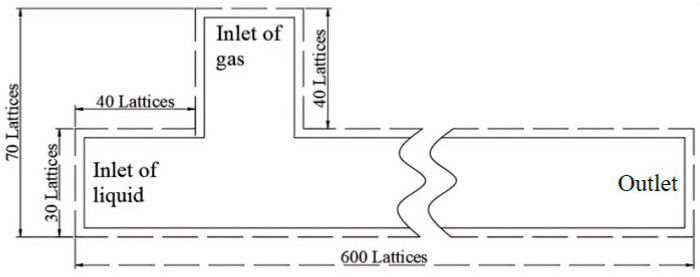
Microchannel model.

Various microchannels generate bubbles, with the T-type microchannel being the most effective. This study simulates two-phase flow in a T-type microchannel at a low capillary number. The continuous phase flows as the primary fluid through the main channel, while the dispersed phase forms tiny bubbles at the intersection. The continuous phase consists of water, silicone oil, alcohol, glucose, and DDB (dodecylbenzene), which flow through the main channel, as illustrated in Figure 1. The gas phase enters through the inlet, serving as the dispersed phase. The two phases converge and flow out of the right exit of the channel.

Calculations are performed in LBM using lattice units and, therefore, need to be converted to macroscopic physical units; in this paper, a 300 µm wide microchannel is simulated using a computational domain with a width of D = 30 lattice points and a length of L = 600 lattice points. Take the air–water system as an example in this paper. The physical properties of the substance are adopted as the value at 20 °C: in this paper, the channel width H = 0.003 m, the viscosity υ = 10^−6^ m^2^/s, and the density ρ = 1000 kg/m^3^, defining the channel lattice width $\tilde{H}=30$, then the length conversion coefficient $C_{H}=10^{-4}\;m$, defining the lattice density $\tilde{\rho }=1$, then the density conversion coefficient $C_{\rho }=1000{\;\mathrm{kg}/m}^{3}$, and defining the relaxation time to be 0.8, then the lattice viscosity is computed $\tilde{\nu }=0.1$, and the viscosity conversion coefficient, so the velocity conversion coefficient is used to convert to macro physical units. The velocity conversion coefficient $C_{u}=C_{\nu }/C_{H}=0.001\;m/s$, and the time conversion $C_{\nu }=10^{-7}\;m^{2}/\;s$ coefficient $C_{t}{=}_{H}/C_{u}=0.1\;s$. The conversion coefficients of the remaining units can be calculated according to the Π theorem in the macroscopic system.

### 2.5. Model Validation

In this section, we perform static validations for Laplace’s law, the contact angle, and thermodynamic consistency for the constructed flow model. Since the kinematic viscosity in the Shan–Chen (S-C) method is controlled via the relaxation time, interface capture does not require a tracking function when handling multiphase flows. The interface evolves naturally without the need for mesh-independence validation. To verify Laplace’s law under static conditions, simulations are conducted to model gas-encapsulated droplets in a periodic, gravity-free domain. Surface tension is calculated using Laplace’s law by confirming that the pressure difference across the phase interface is inversely proportional to the radius of the droplet, with the slope of the resulting line representing the surface tension. Laplace’s law can be expressed as Equation (16):

(16) $$\Delta p=\frac{\lambda }{R}$$

**Figure 2:**
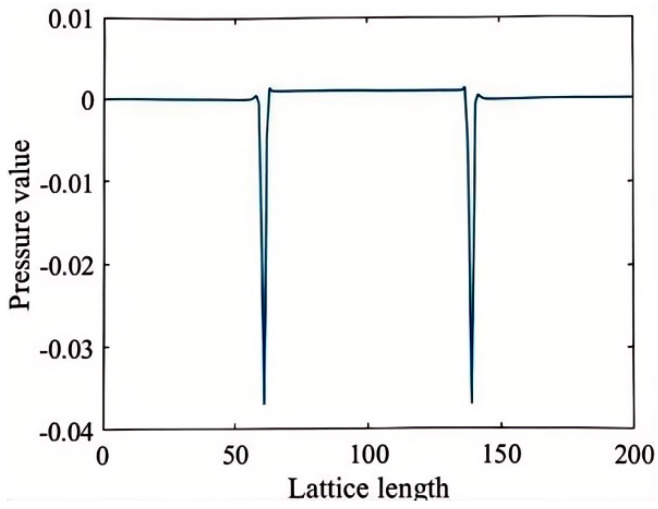
Pressure distribution along the droplet centerline.

∆p represents the pressure difference between the interior and exterior of the droplet, λ denotes the surface tension, and R is the droplet’s radius. The parameter gama is a control parameter within the MRT-Guo model used to adjust the model’s performance but lacks physical significance. In the LBM model, gama is inversely related to the surface tension λ. In this study, droplets of varying radii are placed at the center of a 400 × 200 rectangular domain for simulation. After the model stabilizes, the differential pressure at the phase interface is measured, and the distribution of the pressure difference inside and outside the droplet is shown in Figure 2.

**Figure 3:**
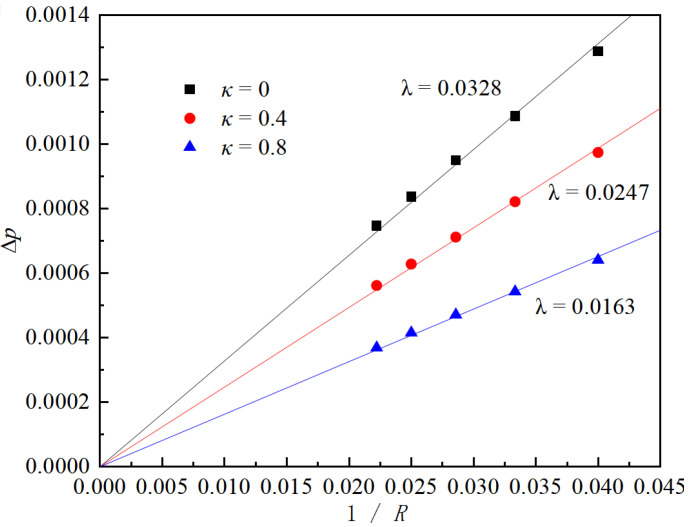
Verification of Laplace’s law.

This study sets T/Tc = 0.5, and the droplet radius is adjusted in a gravity-free field. The system is run for 40,000 steps until it reaches a steady state; at this point, the differential pressure at the phase interface is measured. The Y-value at 100 lattice units in Figure 2 is taken as the internal pressure of the droplet, while the Y-value at 30 lattice units is considered the external pressure. Adjusting κ allows the model’s surface tension to be independently tuned over a broad range, ensuring that the differential pressure maintains a strong linear relationship with the inverse of the droplet radius, as shown in Figure 3.

**Figure 4:**
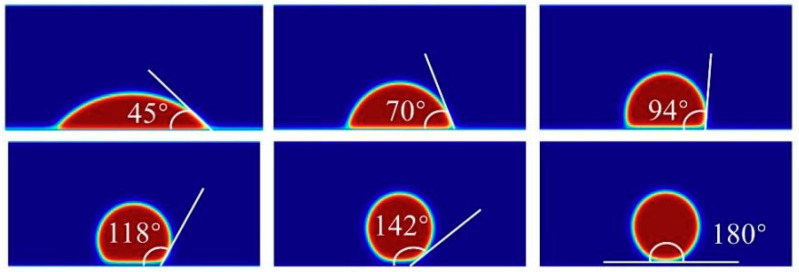
Contact angle verification.

The second part involves the validation of the contact angle, which is related to the fluid–solid wall interaction force—a phenomenon commonly observed in LBM multiphase flow simulations of microflows. In this study, the Ψ-based correction model proposed by Li et al. [33] is employed, using the alcohol–air system as a case study. Droplets with a radius of 30 are placed in a 300 × 200 computational domain, with their center positioned near the lower wall’s central axis (200, 20). The remaining region is filled with the gas phase, with T/Tc = 0.5. The relaxation time serves as the unit for converting the relevant quantities. The simulation results shown in Figure 4 demonstrate that the model can achieve various contact angles, validating its capability to simulate wettability accurately.

**Figure 5:**
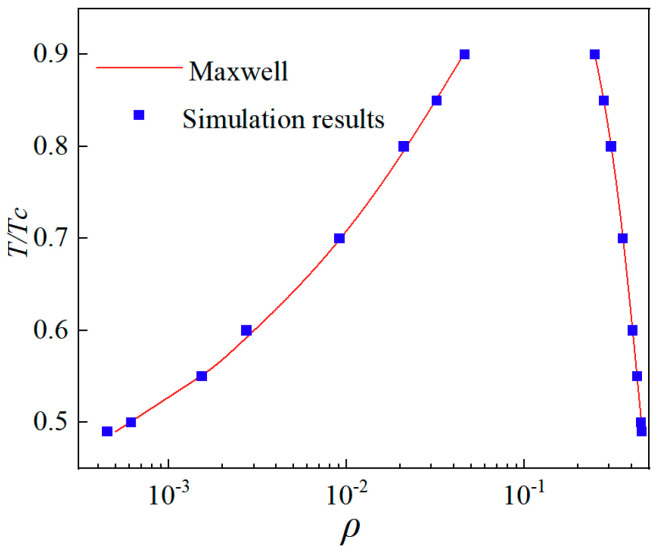
Verification of thermodynamic consistency.

To achieve and validate thermodynamic consistency in this study, σ = 0.125 and β = 1.52 are chosen. A single relaxation scheme simulates a static droplet with a radius 30 in a domain of 200 × 200. The thermodynamic consistency results shown in Figure 5 agree with Li et al.’s findings [29]. This study achieved a density ratio of approximately 700. Additionally, introducing the interface thickness control parameter z modifies the pressure term in the practical density expression: PEOS′ = z × PEOS, where z ranges from 0 to 1 and is inversely proportional to the interfacial thickness. This parameter significantly reduces spurious velocities in the model.

### 2.6. Simulation Results and Analysis

**Figure 6:**
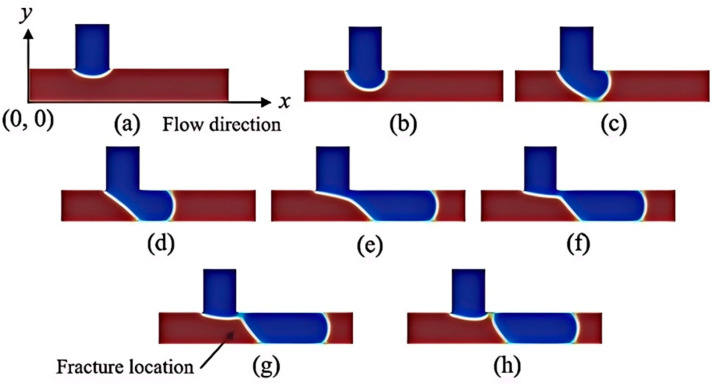
Bubble formation process, red represents the liquid phase and blue represents the gas phase, (**a**–**c**) are the gradual expansion of the gas phase, (**d**–**f**) are the collapse of the bubble within the main channel, (**g**,**h**) are the bubble break-up at the junction.

In this study, the range of simulation parameters is determined based on the characteristics of the collected data. The maximum capillary numbers for five systems—air–water, air–alcohol, air–kerosene, air–glucose, and air–DDB are calculated to be 0.0029, 0.0018, 0.0040, 0.0028, and 0.0049, respectively. The process of bubble formation illustrated in Figure 6 follows the air–liquid elastoplastic flow formation mechanism proposed by Garstecki et al. [10]. Since this study’s simulation capillary number is relatively small, the extrusion mechanism governs the bubble formation. In this regime, interfacial forces dominate over shear stresses, forming immiscible segment plugs, which increase the pressure upstream of the droplet and squeeze the bubble neck.

The process of bubble formation can be divided into three distinct stages:

①. Expansion Stage Figure 6a–c: During this stage, the gas phase continues to expand, progressively cutting off the liquid phase in the horizontal channel, causing the liquid phase to become discontinuous. The gas–liquid interface is convex toward the liquid side, and the stage concludes when the gas phase fully occupies the main channel.

②. Collapse Stage Figure 6c–f: As the flow channel becomes blocked, the liquid phase exerts pressure on the gas phase, causing the gas–liquid interface to gradually convex toward the gas phase. This interface moves downstream, following the gas phase flow, while the area occupied by the gas phase in the horizontal channel continuously increases.

③. Fracture Stage Figure 6f,h: In this stage, the gas–liquid interface breaks near the downstream endpoint of the intersection between the vertical inlet channel and the horizontal channel (see Figure 6h), resulting in the formation of a complete bubble in the horizontal channel. Unlike the previous two stages, the fracture process occurs rapidly, and once completed, the next cycle of bubble development begins.

The gas–liquid two-phase flow rate ratio primarily influences the bubble length when the capillary number is below 0.01. While the gas–liquid flow rate ratio plays a significant role in controlling bubble size, the gas phase flow rate also directly affects the bubble formation process. However, after multiple simulation iterations, it was found that adjusting the gas phase flow rate while keeping other flow parameters constant has minimal impact on bubble size. This finding aligns with the conclusions of Warnier [34]. Therefore, increasing the gas phase flow rate in the simulation is unnecessary to enhance bubble uniformity.

Furthermore, the simulation results indicate that bubble generation is more uniform when the surface tension (σ) decreases and the kinematic viscosity (μ) increases. According to Peng [16], lowering σ can mitigate pressure fluctuations caused by changes in the interface, while increasing μ can enhance the inlet pressure stability against external disturbances. Therefore, relatively lower σ and higher μ contribute to improved bubble uniformity.

A valid LBM model has been established based on the T-type microchannel gas–liquid two-phase flow model developed in this paper. However, manually adjusting the model’s control parameters to identify conditions that stabilize the elastic flow in LBM simulations requires numerous iterations, making pinpointing the optimal model parameters challenging. Machine learning techniques offer a more efficient approach by modeling the elastostatic flow process data and identifying the optimal stability parameter combinations, bypassing the need for complex kinetic theory. The following section details the construction of the machine learning model.

## 3. Machine Learning and Multi-Objective Optimization Model Construction

BP (backpropagation), SVR (support vector regression), and RF (random forest) are three commonly used machine learning models. Since the LBM simulation process involves multiphase field coupling, the nonlinear nature of the simulated data, and a small sample size, the BP model with good generalization ability is chosen to apply the learning results to new knowledge effectively; SVR involves the case of uncertainty in the output of the model; and RF performs exceptionally well on small and medium-sized datasets [35,36]. According to [37,38], the BP, SVR, and RF model characteristics can be summarized in Table 1.

**Figure 7:**
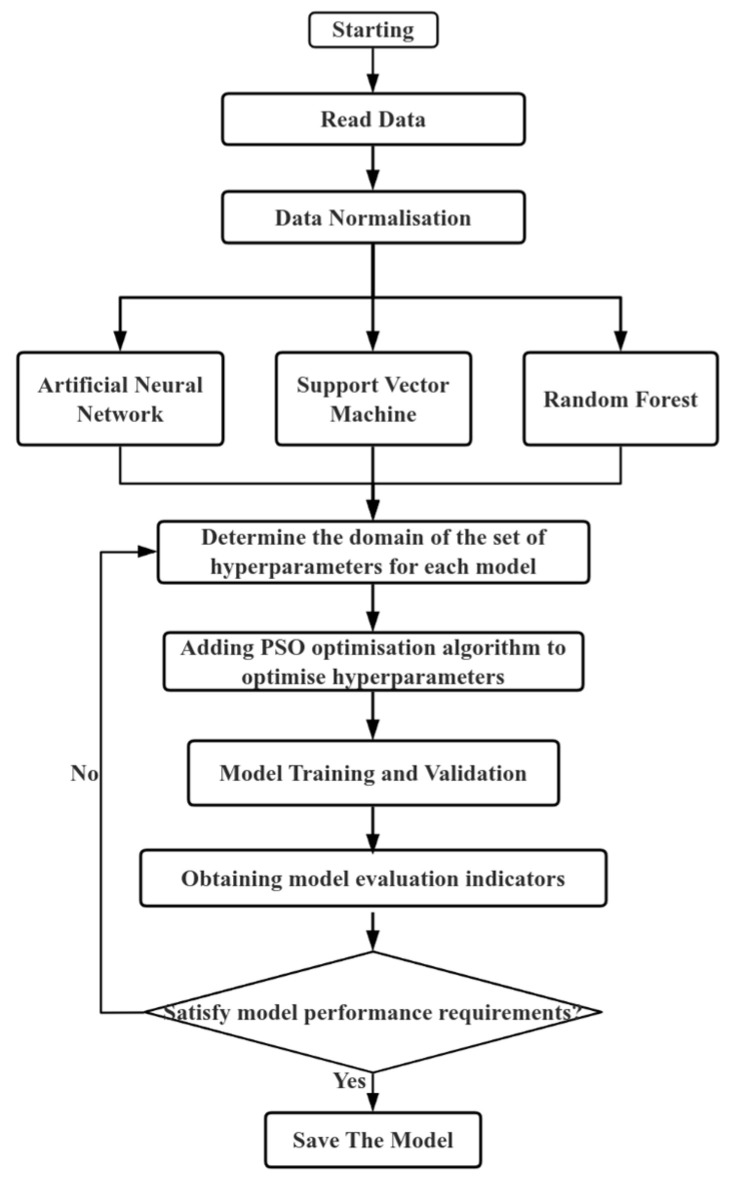
Machine learning modeling flowchart.

This study’s machine learning model is constructed by first establishing the input and output targets. The raw data are obtained through LBM simulations to determine the inputs and outputs. The data are subsequently pre-processed, and BP, SVR, and RF models are selected for modeling. To further enhance the performance of the models, the particle swarm algorithm is incorporated to optimize the model parameters. Following the training process, the prediction results of the machine learning models are evaluated. Models not meeting the evaluation criteria are retrained and validated until they satisfy the required performance standards. This iterative process ensures the selection of the most suitable machine learning model for gas–liquid two-phase flow under low capillary number conditions in T-type microchannels. The specific process is outlined in Figure 7:

### 3.1. Acquisition of Modeling Data

This study aims to establish a gas–liquid two-phase flow model for low capillary number conditions in T-type microchannels, investigate the factors influencing the elongation rate, and identify the optimal model parameters for the desired flow conditions.

#### 3.1.1. Determining the Model Output Elongation

In this study, bubble stability is quantified by the elongation rate θ, which is defined as the ratio of the difference in the lengths of two consecutively occurring bubbles to the length of the second one of them, as shown in Equation (17), where ΔL is shown in Equation (18):

(17) $$\theta =\frac{\Delta L}{L_{2}}$$

(18) $$\Delta L=L_{1}-L_{2}$$

**Figure 8:**
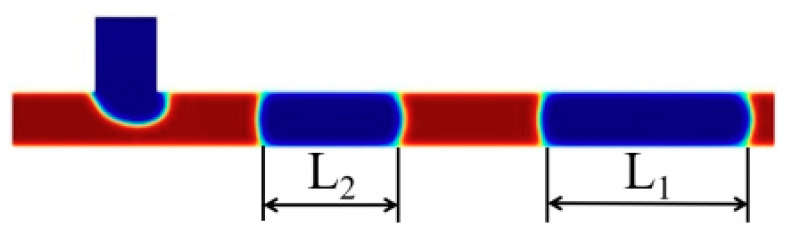
Schematic diagram of the length of the bubbles, red represents the liquid phase and blue represents the gas phase.

L_1_ and L_2_ are the lengths of the first and second bubbles distributed in the T-shaped microchannel at the exact moment, respectively, as shown in Figure 8:

#### 3.1.2. Determining Model Inputs

A review of the literature on bubble homogeneity identified several factors influencing bubble length, as discussed by Peng [16], Garstecki [10], Ganapathy [6], and Dai et al. [39]. These factors include the continuous phase flow rate, dispersed phase flow rate, two-phase flow rate ratio, surface tension, and two-phase viscosity. Studies involving non-Taylorian flows and those focused on special reaction conditions, simulations, and experiments were excluded from this review. In this study, the range of simulation parameters is determined based on the characteristics of the collected data. The input variables are constrained to a low capillary number regime, specifically a two-phase flow rate below 0.006, surface tension parameter γ in the range of 0.11–0.14, continuous phase viscosity between 0.6 and 0.8, and dispersed phase viscosity ranging from 1.25 to 2.26. The lattice Boltzmann method (LBM) generated one hundred and thirty-seven simulation datasets within these parameter bounds. Of these, 80% were randomly allocated to the training set, while the remaining 20% were used as the test set. Each simulation took approximately 2 h to complete. A subset of the simulation data from the 137 sets is presented in Table 2.

#### 3.1.3. Data Pre-Processing

After identifying the flow parameters that influence bubble length through correlation analysis, simulations were conducted by adjusting these parameters within specified ranges. To mitigate the effects of varying data magnitudes and enhance the generalization ability of the model, the data were normalized prior to modeling [33]. The normalization formula used is presented in Equation (19):

(19) $$x_{i}^{*}=\frac{x_{i}-x_{min}}{x_{max}-x_{min}}$$

where $x_{i}^{*}$ denotes the data after normalization, x_i_ denotes the original sample data, and x_max_ and x_min_ denote the maximum and minimum values of the original sample dataset, respectively. The normalized data patterns are introduced into the three machine learning models as dataset modeling.

### 3.2. PSO-BP, PSO-SVR, and PSO-RF Model Hyperparameter Selection

The PSO algorithm is a group intelligence-based optimization method that uses information sharing among particles to drive the evolutionary process. Each particle adjusts its position and velocity based on its own and neighboring particles’ experiences to find the global optimum [40]. The algorithm evaluates populations using regression analysis and terminates when the optimal population’s compatibility rate meets the stopping criterion [41]. PSO is widely used in chemical systems, mechanical designs, and machine learning models.

#### 3.2.1. PSO-BP Model

In this study, the machine learning model has five input nodes corresponding to five parameters, a single hidden layer with three nodes optimized by PSO, and a single output node predicting elongation—Table 3, Table 4 and Table 5 present the optimized hyperparameters and initial weights.

#### 3.2.2. PSO-SVR Model

In this paper, we optimize the loss function by incorporating a penalty factor ccc in the loss function with the parameter g of the gama function in the radial basis kernel function (RBF kernel). This optimization is achieved using the particle swarm optimization (PSO) algorithm, improving training performance. The optimized values of the hyperparameters are shown in Table 6.

#### 3.2.3. PSO-RF Model

Random Forest (RF) is an ensemble learning algorithm that builds multiple decision trees to enhance model accuracy and robustness. By using Bagging (Bootstrap Aggregating) and random feature selection during training, RF generates predictions through either majority voting (for classification) or averaging (for regression). RF is known for its high accuracy, resistance to overfitting, and ability to handle high-dimensional datasets. Additionally, it is robust to missing values and noise, making it effective for complex data. While RF offers strong interpretability and generalizability, it comes with the trade-off of high computational costs and limited adaptability to certain data types. The optimized hyperparameters for the RF model are provided in Table 7.

### 3.3. Multi-Objective Optimization Model Construction by Non-Dominated Sorting Genetic Algorithm

**Figure 9:**
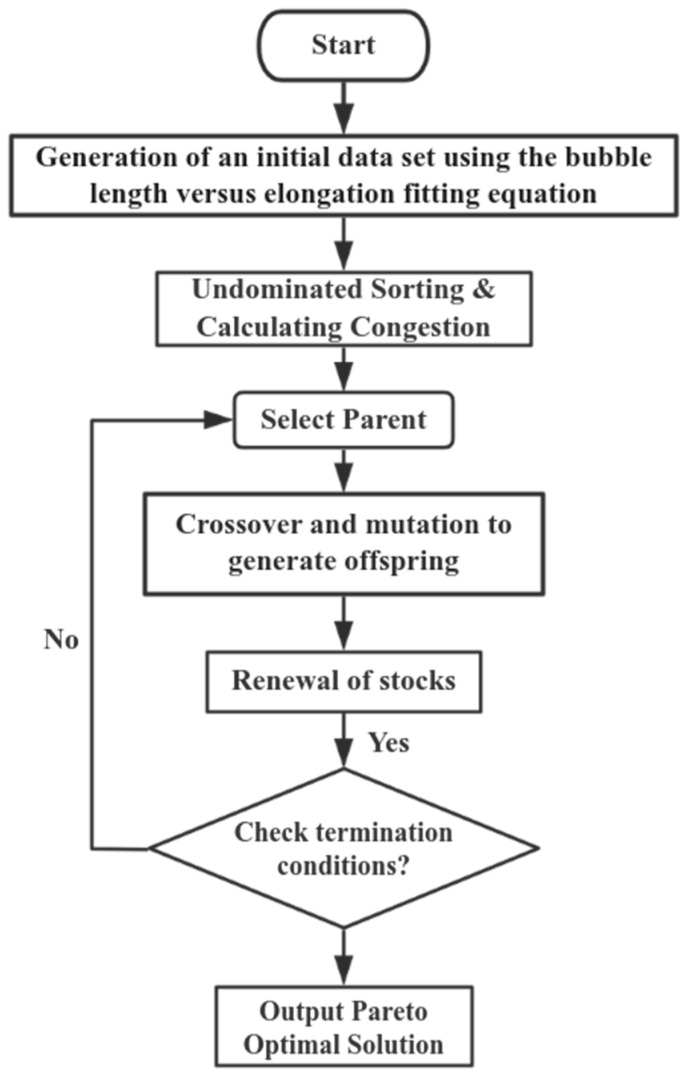
Algorithm flowchart.

The non-dominated sorting genetic algorithm (NSGA) is a multi-objective optimization technique designed to solve optimization problems with conflicting objectives. It introduces the concept of ‘non-dominated sorting’ and does not rely on artificially set objective weights. Instead, the algorithm automatically weighs the objectives through a Pareto dominance relation. By employing non-dominated ordering and congestion distance computation, NSGA effectively maintains the diversity of the solution set, avoids premature convergence, resolves conflicts between objective functions, and generates a diverse set of Pareto optimal solutions. The goal of this paper is to obtain uniform and large-size bubbles, and it is not easy to satisfy these two goals at the same time if only the LBM method is used. Therefore, this paper will combine the LBM method with the NSGA-II algorithm, and use the NSGA-II algorithm to find the bubble flow conditions with excellent heat and mass transfer performance and stable flow. Figure 9 shows the specific steps of combining the LBM method with the NSGA-II algorithm:

Since all multi-objective optimization algorithms require an objective function, this paper will use the LBM simulation data to fit the expressions for bubble length, bubble elongation, and related parameters. According to the bubble length calculation formula mentioned in Grasiki as in Equation (20):

(20) $$l_{g}\;/\;D\;=C*Ca^{-1}$$

where D is the channel width, Ca is the capillary number, lg is the bubble length, and C is the coefficient to be determined in this paper after polynomial fitting, obtained C = 0.1082.

In the above section, we analyzed the effects of the inlet flow rate, liquid phase viscosity, surface tension, and other factors on the bubble elongation rate of the elastic flow. Peng [16] proposed the bubble elongation and the related factors relationship equation, based on which the elongation rate was established with the liquid phase flow rate, the liquid phase viscosity, the surface tension, and the gas phase flow rate of the fitted relationship, on the bubble elongation θ after the fitting to obtain Equation (21) as follows:

(21) $$\theta =\frac{\sqrt{2}c(1)\sigma (Q(1)/Q(2))/c(2)+c(3)c(5)(\sigma /c(4))}{c(7)(c(6)+c(1)(Q(1)/Q(2)))(Q(1)\mu /c(2)/c{\left(4\right)}^{3})+c(8)(\sigma /c(4))c(5)+c(2)(2/c(2)+2/c(4))}$$

where Q1 and Q2 represent the liquid and gas phase velocities, respectively, σ represents the surface tension, and μ is the liquid phase viscosity after polynomial fitting; the values were obtained as shown in Table 8, respectively:

To verify the accuracy of the fitting formula, it was substituted into the simulated working conditions to solve the elongation, and the fitting results were obtained. After the comparison in Table 9, it is found that the fitting formula has a high accuracy, and the difference between the results is less than 2%.

Different factors, such as gas–liquid velocity and liquid viscosity, influence the bubbles in various ways, and these factors are interrelated in the flow. To determine the optimal flow conditions in the T-channel elastic flow, a non-dominated sorting genetic algorithm (NSGA) is applied based on the fitting relationship discussed earlier. The objective is to maximize the bubble length (lg) while minimizing the elongation ratio (θ). This optimization problem can be expressed by Equation (22):

(22) $$\begin{array}{l} \min F(Q(1),Q(2),\sigma ,\mu )=[1/\theta (Q(1),Q(2),\sigma ,\mu ),1/l_{g}(Q(1),Q(2),\sigma ,\mu )]\\ s.t.\theta (Q(1),Q(2),\sigma ,\mu ),l_{g}(Q(1),Q(2),\sigma ,\mu )\geq 0 \end{array}$$

In this optimization problem, Q(1) represents the liquid flow rate, Q(2) represents the gas flow rate, σ represents the surface tension, and μ represents the liquid viscosity. The objective optimization functions are θ(Q(1), Q(2),σ,μ) and l_g_(Q(1), Q(2),σ,μ). As discussed earlier, there is a conflict between the two objectives, meaning that no single globally optimal solution satisfies both objectives simultaneously. Consequently, we obtain the Pareto fronts for velocity, viscosity, and surface tension.

Table 10 shows the specific parameters for applying the NSGA-II algorithm application:

In this paper, the initial data for multi-objective optimization are generated by LBM simulation, and the low capillary number fluid problem involves a nonlinear, multi-physical field coupling problem, so the simulation is time-consuming, with an average of 2 h each time. The simulation data are used to generate the fitting function as the objective function of the NSGA-II algorithm, so the single evaluation time is shorter. The standard time complexity of the NSGA-II algorithm is O(MN^2^); M represents the number of objective functions, and N represents the population size. In this paper, the objective function is 2, the population size N is set to 100, the multi-objective optimization process is iterated for 100 times, and the multi-objective optimization process consumes time in total 30 s.

Regarding whether the algorithm suffers from premature convergence, as demonstrated by Deb [42] (2002), the combination of non-dominated ordering and congested distance assignment in NSGA-II inherently prevents premature convergence by balancing convergence and diversity. In this paper, we incorporate a cross-variance strategy, where the population size (N = 100) and the mutation probability (pMutation = 0.4%) are carefully tuned to ensure that the search space is fully explored while maintaining convergence efficiency.

### 3.4. Indicators for Model Evaluation

The accuracy of the developed model is measured in terms of three metrics: goodness-of-fit (R^2^), root mean square error (RMSE), and mean absolute error (MAE) on the test sample set, as shown in Equations (23)–(25).

(23) $$R^{2}=1-\frac{\sum_{i=1}^{n}{\left(y_{i}-f_{i}\right)}^{2}}{\sum_{i=1}^{n}{\left(y_{i}-\bar{y}\right)}^{2}}$$

(24) $$RMSE=\sqrt{\frac{\sum_{i=1}^{n}{\left(y_{i}-f_{i}\right)}^{2}}{n}}$$

(25) $$MAE=\frac{\sum_{i=1}^{n}\left|y_{i}-f_{i}\right|}{n}$$

where y_i_ and f_i_ denote the target and predicted values of the ith sample, respectively. Where R^2^ is used to evaluate the model fitting effect, the better the model fitting effect, the closer R^2^ is to 1. RMSE measures the deviation between the predicted and real values, which is more sensitive to outliers; MAE is used to evaluate the prediction accuracy. The higher the accuracy, the closer MAE is to 0.

## 4. Results and Discussion

### 4.1. Comparison of the Accuracy of the Three Models

For the T-type microchannel gas–liquid two-phase flow LBM simulation data, 80% of the dataset (i.e., 110 groups) is randomly selected as the training set. The PSO-SVR, PSO-BP, and PSO-RF models, as established in Section 2.2, are then used for modeling. The predicted values obtained from these models are compared with the real values derived from the LBM numerical simulation.

#### 4.1.1. Comparison of Train Set Accuracy

**Figure 10:**
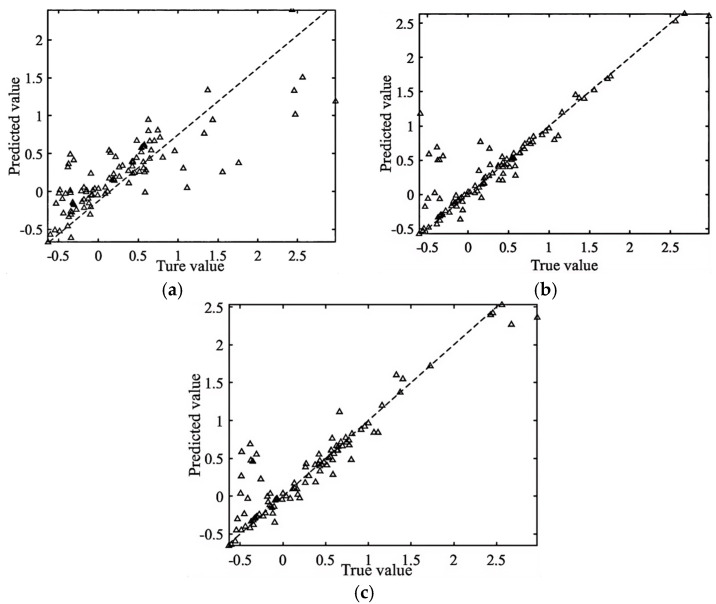
Correlation between predicted and true values of the training set using three different models. (**a**) PSO-SVR model Coefficient of determination R^2^ = 0.62, (**b**) PSO-BP model Coefficient of determination R^2^ = 0.80, (**c**) PSO-RF model Coefficient of determination R2 = 0.85.

In total, 80% (i.e., 110 groups) of the T-type microchannel gas–liquid two-phase flow LBM simulation data were randomly selected as the training set, respectively, and the results of the training comparison are shown in Figure 10.

#### 4.1.2. Comparison of Test Set Accuracy

**Figure 11:**
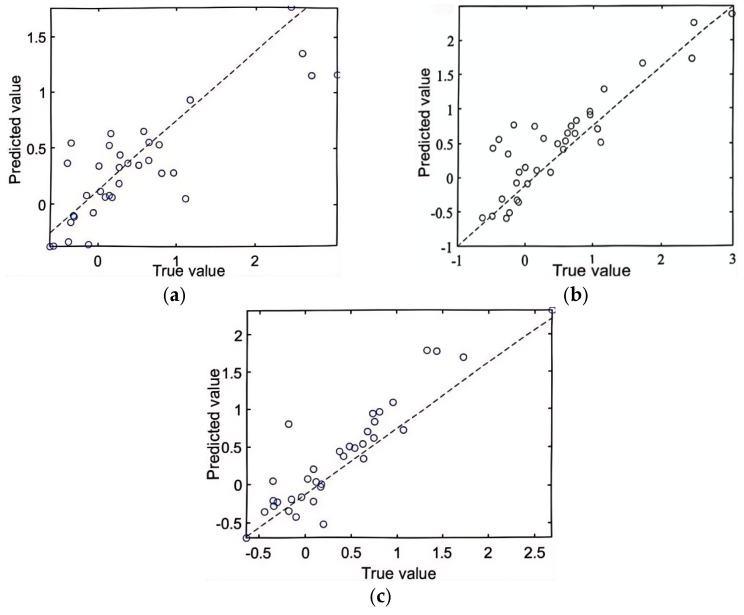
Correlation between predicted and true values of the test set using three different models. (**a**) PSO-SVR model coefficient of determination R^2^ = 0.61, (**b**) PSO-BP model coefficient of determination R2 = 0.81, (**c**) PSO-RF model coefficient of determination R2 = 0.84.

20% (i.e., 27 sets) of the T-type microchannel gas–liquid two-phase flow LBM simulation data were randomly selected as the test set, respectively, and the comparison of the test results is shown in Figure 11.

Comparing the test set with the training set of the three machine learning models, it is found that the coefficient of determination of the training set is almost the same as that of the test set, and hence, there is no overfitting in the training model. The PSO-BP and the neural network models have a lower R^2^ value due to the limited number of features in the samples and insufficient data in the training set. The PSO-RF model has a higher coefficient of determination than the PSO-SVR model.

Due to the objective limitation of small sample data in this study (the total sample size is only 137 groups), if the training set is too small according to the traditional division, it may lead to underfitting of the model [43] and poor model results. Due to the long simulation time and high time cost of LBM, as a supplement, we use three sets of never-seen data as a test, and the three sets of data are not involved in model training or hyperparameter tuning. The values of the stochastic model parameters are shown in Table 11.

**Figure 12:**
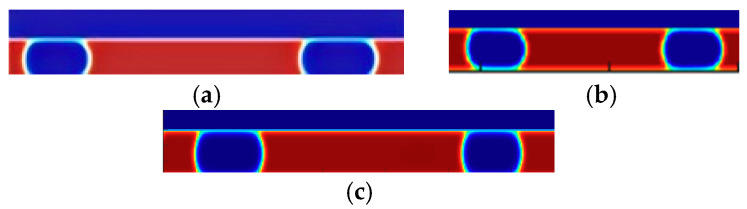
Variation of bubble length with stochastic model parameters. (**a**) The first set of simulation results. (**b**) The second set of simulation results. (**c**) The third set of simulation results. Red represents the liquid phase and blue represents the gas phase.

LBM simulations are performed for the above three sets of stochastic model parameters, and the simulation results are shown in Figure 12, where the flow directions are all from left to right flow:

The bubble length for each group is measured by the lattice length at the first and last points along the centerline of the bubbles, as shown in the simulation results in Figure 11. The elongation rate is then calculated using Equation (10). For example, the predicted elongation rate in the first data group is θ = 10.95%, indicating that the bubble length increases with flow. In contrast, the predicted elongation rate for the third group is θ = −9.43%, indicating that the bubble length decreases with flow.

Three sets of model parameter combinations, as shown in Table 12, were input into the PSO-BP, PSO-SVR, and PSO-RF machine learning models. These models were then used to predict the bubble elongation rate for each set of parameters. The predicted and true elongation values for each of the three machine learning models under the same parameter sets are presented in Table 12.

As shown in Table 12, the PSO-RF model has the smallest prediction error, ranging from 0.24% to 1.18%, and can accurately predict all random situations. In contrast, the PSO-BP model has a maximum prediction error of 6.85%, and the PSO-SVR model has a relatively large maximum error of 8.67%. Through comparative testing of the three typical models, the random forest (RF) model shows excellent prediction performance: its maximum prediction error is only 1.18%, especially in the microchannel flow prediction task, and the error rate is lower than the 2.1% described in the paper when compared to the Deep Neural Network (DNN) model used in the literature [44], which confirms the strong characterization of the microscale flow nonlinear characterization of microscale flow nonlinearities.

**Figure 13:**
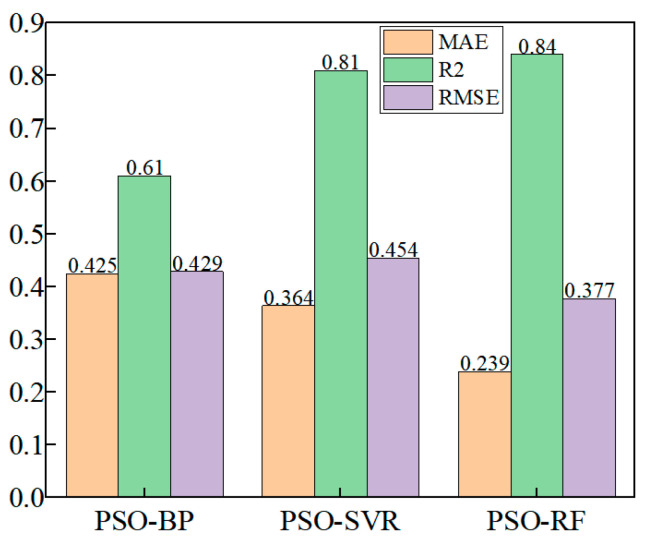
Comparison of the performance of the three machine learning models.

The PSO-RF model achieves a higher coefficient of determination than the PSO-SVR model. The histogram of statistical parameters (R^2^, RMSE, MAE) for different outputs is shown in Figure 13:

The results indicate a positive correlation between the simulated data and the predicted data from the PSO-RF model, with no overfitting observed during the learning stage. The PSO-RF model demonstrates higher prediction accuracy (higher R^2^ and lower RMSE and MAE values) and superior overall performance. This can be attributed to the model’s ensemble learning approach. RF reduces the risk of overfitting and enhances generalization by constructing multiple decision trees and aggregating their predictions through voting or averaging. Compared to SVR, RF does not require the selection of complex kernel functions and is better at capturing nonlinear relationships. Additionally, RF excels at handling high-dimensional data and performs automatic feature selection, which helps reduce the influence of irrelevant features [35]. In contrast, BP neural networks are more prone to local optima. These advantages make RF more robust and accurate in its predictions. After hyperparameter optimization, the PSO-RF model shows a superior training process and noise immunity compared to the other models, especially in handling complex datasets. The PSO-RF model outperforms the PSO-BP and PSO-SVR models in predicting bubble elongation.

### 4.2. Prediction of Optimal Model Parameters for LBM Models

**Figure 14:**
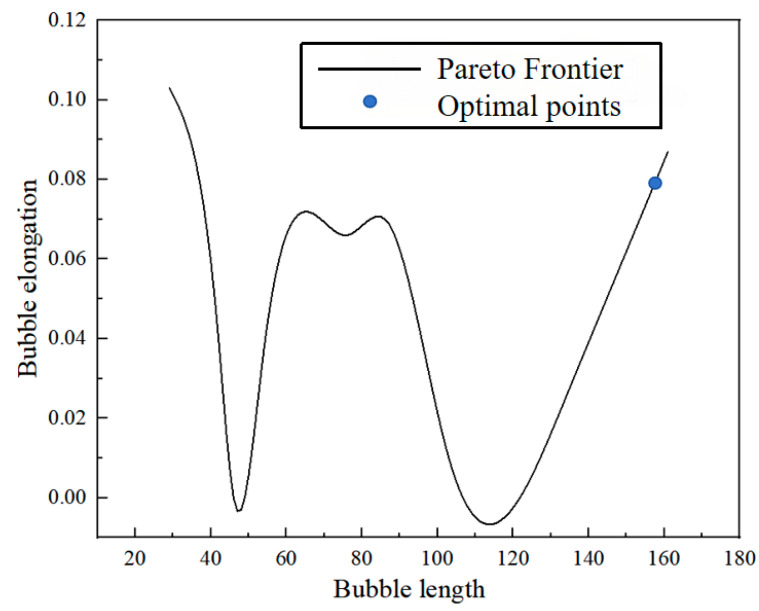
Pareto frontier curve.

According to the fitting equation between bubble length and bubble elongation, the result of the multi-objective optimization problem described by Equation (22) is a curve consisting of a set of Pareto optimal solution points for the gas–liquid velocity condition, which corresponds to the optimal flow transfer performance with the optimal points indicated on the curve, as shown in Figure 14. 

According to the non-dominated genetic algorithm, the solution with the least crowding on the Pareto front is selected as the optimal solution, and the decision variables corresponding to each set of optimal solutions and the bubble length and bubble elongation under this decision variable are shown in Table 13.

Table 13 shows that most optimal solutions are concentrated within the air–water system, where the continuous phase viscosity is 2.2 and the dispersed phase viscosity is 0.6. Additionally, decreasing the continuous phase velocity increases bubble length. The most extended bubble lengths are achieved when the continuous phase velocity is set to 0.0015; the bubble elongation is lower under these conditions. To verify the optimal solution, the predicted results are substituted into the LBM model for simulation.

**Figure 15:**
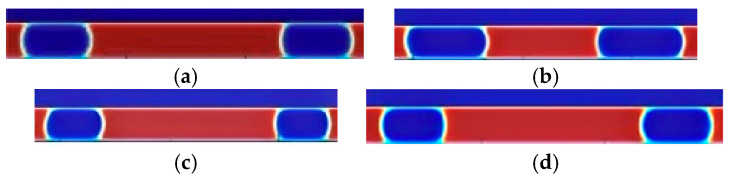
Optimal solution set validation. (**a**) Fourth set of simulation results. (**b**) Group V simulation results. (**c**) Non-optimized results. (**d**) Non-optimized results. Red represents the liquid phase and blue represents the gas phase.

As shown in Figure 15, within the optimal working condition range, the fourth and sixth data groups are selected for simulation based on the optimal conditions from Table 13. Additionally, two other experimental conditions falling outside the optimal working range (non-optimal conditions) are chosen for comparison.

The decision variables for the two sets of non-optimal operating conditions are shown in Table 14.

**Figure 16:**
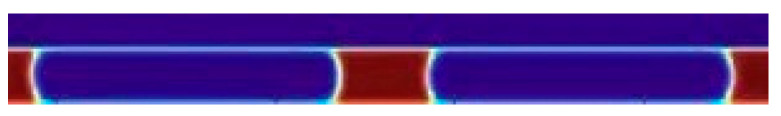
Variation of bubble length with stochastic model parameters. Red represents the liquid phase and blue represents the gas phase.

The comparison graphs demonstrate that the bubble elongation is significantly reduced under optimal conditions, and the flow remains stable; whereas, under non-optimal conditions, the bubble length fluctuates with the flow. To achieve uniform, stable, and long elastic flow bubbles at low capillary numbers, the optimal solution is selected from the Pareto front generated by NSGA-II multi-objective optimization. This solution is then substituted into the LBM elastic flow model for validation, and the resulting data are presented in Figure 16.

The decision variables corresponding to this working condition are shown in Table 15.

Through multi-objective optimization, homogeneous and stable long bubbles were successfully generated in the LBM elastic flow model. Higher heat and mass transfer efficiencies than those of short bubbles have been obtained [1] while improving the stability of the flow in the elastic flow model [4,5]. The optimal solution obtained by applying NSGA-II to the fitted Equation (21) is crucial in enhancing the elastic flow model’s heat and mass transfer efficiency within the microchannel while ensuring precise control over the reaction process.

It is worth noting that the objective function of elastic static flow at a low capillary number is computationally expensive, requiring several hours for a single simulation, resulting in a long overall optimization time, which is limited at the industrial application level. This type of optimization problem is nonlinear and multi-peaked. Although the NSGA-II algorithm used in this paper improves the problem using the congestion distance and the non-dominated ordering, it may still lead to premature convergence in the case of higher parameter dimensions and a larger parameter range. It may still lead to premature convergence. Moreover, the NSGA-II algorithm has many parameters, and adjusting the parameters requires a specific trial-and-error time, so applying this method in the case of low capillary numbers has some limitations.

## 5. Conclusions

In this study of low-capillary elastic flow processes, the lattice Boltzmann method (LBM) establishes the elastic flow model. A machine learning approach combined with particle swarm optimization (PSO) is proposed to address finding optimal flow parameters under low capillary numbers. A multi-objective optimization uses the non-dominated sorting genetic algorithm (NSGA). As a result, a coupled LBM–machine learning–PSO numerical simulation model for multiphase flow is developed. This approach improves the efficiency of heat and mass transfer in elastic flow under low capillary numbers and identifies the optimal model control parameters for the working conditions. The main contributions of this paper are summarized as follows:

① A gas–liquid two-phase flow model for a high gas-to-liquid ratio T-type microchannel is developed based on the lattice Boltzmann method (LBM) theory. The model is validated through static Laplace’s law, contact angle, and thermodynamic consistency tests to confirm its accuracy and reliability.

② A machine learning method incorporating particle swarm optimization (PSO) is proposed to develop a coupled multiphase flow model combining LBM simulations and machine learning. The model is constructed using three machine learning techniques, PSO-SVR, PSO-BP, and PSO-RF, to predict bubble elongation rates under five different sets of flow parameters, aside from the training and test sets. The results indicate that the PSO-RF model outperforms the PSO-BP and PSO-SVR models, achieving an R2 value greater than 0.83 and a mean absolute error (MAE) below 0.24, demonstrating superior prediction accuracy for bubble elongation rates.

③ For the two optimization objectives of bubble length and uniformity, a non-dominated genetic sorting algorithm (NSGA) is employed for multi-objective optimization, resulting in a Pareto optimal solution set. This set determines the optimal flow parameters, achieving a bubble length of 173 lattice units and an elongation rate of θ = 0.079% under low capillary conditions. These optimal parameters ensure a balance between heat and mass transfer efficiency and flow stability, leading to improved control over the flow process.

In this paper, the lattice Boltzmann method (LBM) is utilized to effectively address the issue of elastic flow instability at low capillary numbers, significantly improving both heat and mass transfer efficiency and model stability. The coupled LBM–machine learning–particle swarm algorithm numerical simulation model developed in this study provides valuable insights into the elastic flow characteristics and bubble dynamics in T-type microchannels by optimizing the elastic flow model under these conditions. This model is a powerful tool for studying flow control and heat/mass transfer processes in T-type microchannel reactors.

## Figures and Tables

**Table 1:** Comparison of three machine learning characteristics.

Characterization	BP Neural Network	SVR	RF
nonlinear modeling capabilities	vigorous	vigorous	vigorous
learning and generalization skills	vigorous	vigorous	vigorous
suitability	classification, regression, clustering, etc.	regression, high-dimensional data processing	regression, high-dimensional data processing
training time	longer	comparatively long	shorter
local optimum	vulnerable to	likelihood	lesser
parameter sensitivity	sensitivity	sensitivity	lower sensitivity
applicable data size	broad scale	medium	broad scale
computational complexity	medium	high	high

**Table 2:** T-type microchannel gas–liquid two-phase flow part of LBM simulation data.

Groups	Continuous Phase Velocity	Dispersed Phase Velocity	Gama Value	Continuous Phase Viscosity	Dispersed Phase Viscosity	Elongation/%
1	0.005	0.005	0.13	1.58	0.79	0.302
2	0.004	0.004	0.11	1.65	0.74	0.915
3	0.0015	0.004	0.12	2.2	0.6	0.566
4	0.0025	0.004	0.12	2.2	0.6	0.373
5	0.003	0.004	0.12	2.2	0.6	0.376
6	0.0035	0.004	0.12	2.2	0.6	0.515
7	0.0055	0.0055	0.12	1.55	0.6	−0.545
...	...	...	...	...	...	...
9	0.0035	0.0035	0.12	1.55	0.6	0.662
10	0.006	0.006	0.12	1.55	0.6	−0.231
133	0.005	0.004	0.12	1.55	0.6	0.262
134	0.005	0.005	0.15	1.25	0.8	1.372
135	0.0035	0.004	0.12	1.44	0.6	1.065
136	0.004	0.004	0.13	1.82	0.72	1.76
137	0.005	0.005	0.14	1.44	0.6	0.735

**Table 3:** Hyperparameters of the BP model after particle swarm optimization.

Parameters	Selection Range	Final Value
Number of hidden layer nodes	[3–6]	3
Type of activation function	sigmoid, relu, tanh	sigmoid
Penalty coefficientc	[0.001, 0.01, 0.1]	0.001

**Table 4:** Initial weights after particle swarm optimization: input layer to hidden layer.

	Input Layer Nodes	1	2	3	4	**5**
Hidden Layer Node						
1		2.9297	0.4736	0.2100	1.3039	1.0948
2		0.6342	1.0059	1.0432	0.1369	0.3695
3		0.5965	0.8656	0.8292	0.1283	1.8448

**Table 5:** Initial weights after particle swarm optimization: hidden layer to output layer.

	Hidden Layer Node 1	Hidden Layer Node 2	Hidden Layer Node 3	Output Layer Node
weights	0.4067	1.4686	0.5064	−0.4521

**Table 6:** Hyperparameters of the SVR model after particle swarm optimization.

	PSO-SVR	
Parameters	Selection Range	Final Value
Kernel function type	Linear, polynomial, radial basis functions, sigmoid	sigmoid
gama	[0.0001, 0.001, 0.01, 0.1]	0.01
Penalty coefficientc	[0.001, 0.01, 0.1]	0.01

**Table 7:** Hyperparameters of the RF model after particle swarm optimization.

	PSO-RF	
Parameters	Selection Range	Final Value
n_estimators	[10, 1000]	350
max_depth	[1, 50]	5
min_samples_leaf	[1, 20]	10
min_samples_split	[2, 20]	7

**Table 8:** Coefficient values of the fitted equation for air bubble elongation rate.

c(1)	c(2)	c(3)	c(4)	c(5)	c(6)	c(7)	c(8)
5.1793	0.9616	1.8439	1.2749	1.2545	1.8437	0.2049	0.7857

**Table 9:** Comparison of polynomial fitting results with simulation results.

Groups	Continuous Phase Velocity	Dispersed Phase Velocity	Gama Value	Continuous Phase Viscosity	Fitted Elongation θ/%	LBM Simulated Elongation θ/%	Fitted Bubble Length l_g_	LBM Analogue Bubble Length l_g_
1	0.005	0.004	0.12	1.8	13.1	13.3	46	45
2	0.005	0.005	0.11	1.65	−12.4	−11.9	45	47
3	0.003	0.005	0.12	1.94	40.8	42.2	81	83

**Table 10:** NSGA-II algorithm-specific parameter settings.

Parameter	Symbol/Variable	Value/Expression	Description
Maximum Iterations	MaxIt	100	The maximum number of iterations for the algorithm.
Population Size	N	100	The number of individuals in each generation.
Crossover Percentage	pCrossover	0.7%	The proportion of offspring generated by crossover operations.
Number of Offspring	nCrossover	36	The number of offspring generated by crossover.
Mutation Percentage	pMutation	0.4%	The proportion of mutants generated by mutation operations.
Number of Mutants	nMutation	20	The number of mutants generated by mutation.
Mutation Rate	mu	0.02	The probability of mutation operations.
Mutation Step Size	Sigma	[0.0005, 0.0005, 0.003, 0.095]	The step size used in mutation is related to the variable range.
Number of Variables	nVar	4	The number of decision variables in the optimization problem.
Lower Bound	VarMin	[0, 0, 0.11, 1.25]	The minimum value for each decision variable.
Upper Bound	VarMax	[0.005, 0.005, 0.14, 2.2]	The maximum value for each decision variable.

**Table 11:** Parameters of the five-group stochastic model.

Groups	Continuous Phase Velocity	Dispersed Phase Velocity	Gama Value	Continuous Phase Viscosity	Dispersed Phase Viscosity	Elongation θ/%
Group I	0.0038	0.0038	0.12	2.2	0.6	10.95
Group II	0.0035	0.004	0.13	1.68	0.68	2.12
Group III	0.005	0.005	0.11	1.94	0.7	−9.43

**Table 12:** Comparison of predicted elongation with simulated true elongation for PSO-SVR, PSO-RF, and PSO-BP models.

Groups	PSO-BP		PSO-SVR		PSO-RF		LBM Simulated Elongation/%
	Predicted Elongation θ/%	Tolerance Value/%	Predicted Elongation θ/%	Tolerance Value/%	Predicted Elongation θ/%	Tolerance Value/%	
Group I	8.87	2.08	7.44	3.51	9.77	1.18	10.95
Group II	5.51	3.39	10.79	8.67	1.88	0.24	2.12
Group III	−16.28	6.85	−6.46	2.97	−9.11	0.32	−9.43

**Table 13:** Optimal solution set.

Groups	Continuous Phase Velocity	Dispersed Phase Velocity	Gama Value	Continuous Phase Viscosity	Bubble Elongation θ	Bubble Length l_g_
1	0.006	0.006	0.12	2.2	0.103	29
2	0.005	0.005	0.12	1.8	0.04	42
3	0.005	0.005	0.12	2.2	0	46
4	0.005	0.005	0.13	1.55	0.037	54
5	0.0035	0.005	0.12	1.55	0.066	76
6	0.003	0.005	0.12	2.2	0.069	87
7	0.0025	0.005	0.12	1.44	0.01	103
8	0.002	0.005	0.12	2.2	0.023	133
9	0.0015	0.005	0.12	2.2.	0.087	161

**Table 14:** Non-optimal working condition parameters.

Groups	Continuous Phase Velocity	Dispersed Phase Velocity	Gama Value	Continuous Phase Viscosity	Dispersed Phase Viscosity	Elongation θ/%
c	0.0046	0.0046	0.12	1.55	0.6	−13.43
d	0.005	0.005	0.13	1.55	0.6	15.38

**Table 15:** Optimal working conditions for LBM elastic flow model under NSGA-II prediction.

Continuous Phase Velocity	Dispersed Phase Velocity	Gama Value	Continuous Phase Viscosity	Dispersed Phase Viscosity	Bubble Elongation θ	Bubble Length l_g_
0.00156	0.005	0.121	2.2	0.6	0.079	160

## Data Availability

The original contributions presented in this study are included in the article. Further inquiries can be directed to the corresponding authors.
